# Point-of-care Ultrasound Trumps Computed Tomography in a Case of Abdominal Aortic Aneurysm Assessment

**DOI:** 10.7759/cureus.5989

**Published:** 2019-10-24

**Authors:** Lara N Goldstein, Mike Wells

**Affiliations:** 1 Emergency Medicine, University of the Witwatersrand, Johannesburg, ZAF

**Keywords:** abdominal aortic aneurysm, computer tomography, point of care ultrasound

## Abstract

Patients who present to the emergency department (ED) with aortic emergencies can be some of the highest acuity patients that we manage. Ultrasonography performed at the bedside is traditionally considered to be a screening test that is especially useful in the unstable patient. Computed tomography (CT) with angiography is the imaging modality of choice to confirm the diagnosis and plan the management of abdominal aortic aneurysm (AAA), as an ultrasound is generally thought not to provide the clinician with sufficient anatomical information. We present a case of a patient with an abdominal aortic aneurysm where evidence obtained from the ultrasound provided more useful information regarding aneurysm structure and stability than did CT.

## Introduction

Patients who present to the emergency department (ED) with aortic emergencies (abdominal aortic aneurysm (AAA) and/or aortic dissection) can be some of the highest acuity patients that we manage [1]. Although a pulsatile abdominal mass may be palpable on physical examination, diagnostic testing is required to confirm or refute the diagnosis [1]. In the ED, bedside ultrasonography is readily available to evaluate a patient with suspected AAA, however, computed tomography (CT) is generally considered to be the diagnostic modality of choice for the AAA to be characterized and delineated for definitive surgical management [1].

## Case presentation

An 86-year-old man was referred to our ED by his general practitioner. He complained of fatigue, lower back pain, mild abdominal discomfort, and pre-syncope. He was an ex-smoker, a long-standing, well-controlled hypertensive patient on an angiotensin-converting enzyme inhibitor who had otherwise been “well.”

Besides relative hypotension (blood pressure measured 106/58 mmHg in both arms) in the ED, the rest of the patient’s vital signs were within normal limits (heart rate 86 beats per minute, respiratory rate 20 breaths per minute, oxygen saturation 94% on room air). There was mild abdominal tenderness, but no pulsatile mass was detected. The rest of the examination was non-contributory. 

A urine dipstick showed a trace of blood. Venous blood gas showed signs of mild hypoperfusion (base excess -6 mmol/L) and a slightly elevated lactate level (3 mmol/L). His hemoglobin was normal.

**Figure 1:**
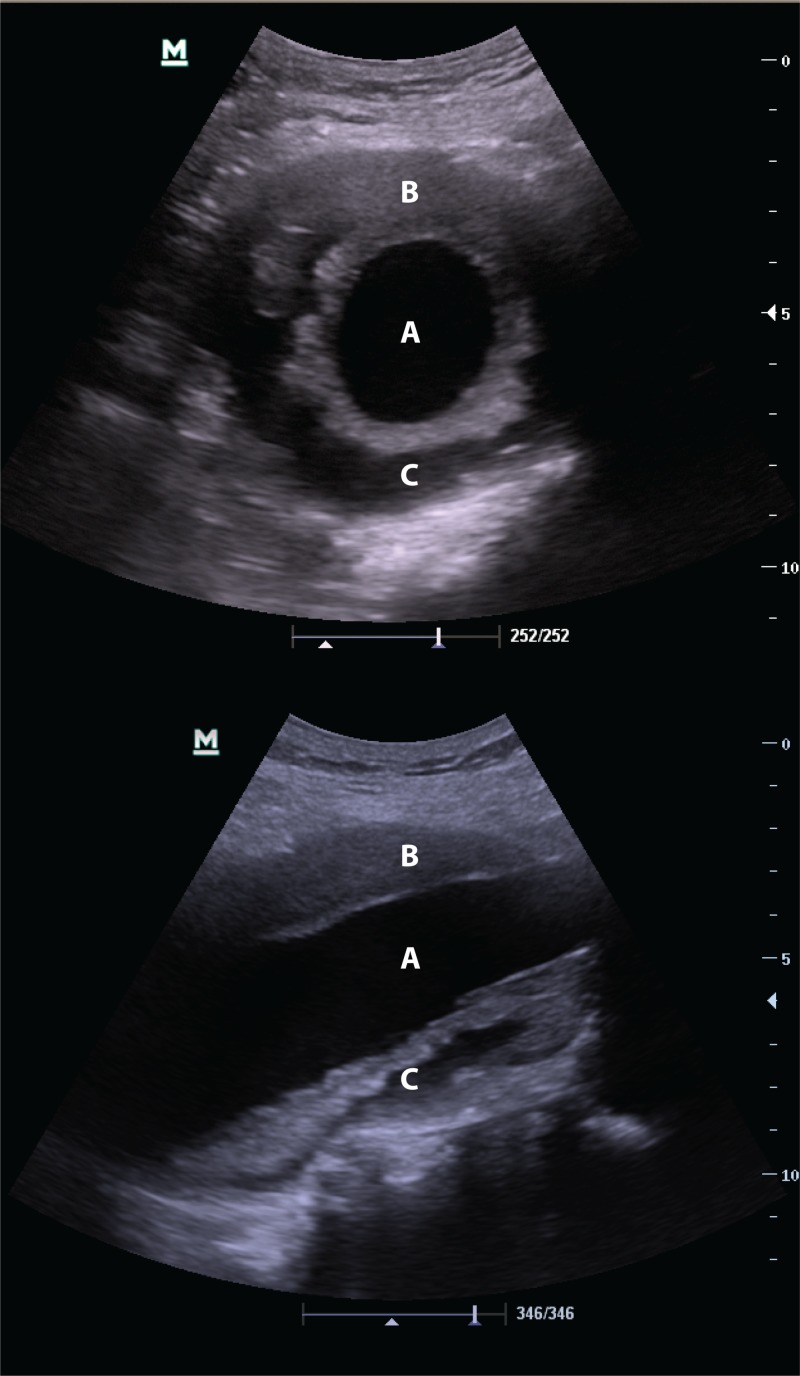
Point-of-care ultrasound images of the abdominal aorta Top: transverse section; Bottom: longitudinal section Both the transverse and longitudinal views of the abdominal aorta show a large abdominal aortic aneurysm with a central lumen (A), an area of stable thrombus (B), and areas of dissection or hemorrhage within the thrombus in the lateral and posterior aspects of the aorta (C).

A bedside ultrasound was performed by the on-duty emergency physician with the portable Mindray M7 C5-2s adult convex probe (Mindray Medical International Limited, Shenzhen, China). It revealed a large 8 x 9cm infrarenal AAA (Figure 1). There were heterogeneous areas of fresh hemorrhage and/or dissection within the aneurysmal thrombus structure: a very unstable condition, as bleeding or dissection into the intraluminal thrombus in abdominal aortic aneurysms is associated with rupture [2-3].

**Figure 2:**
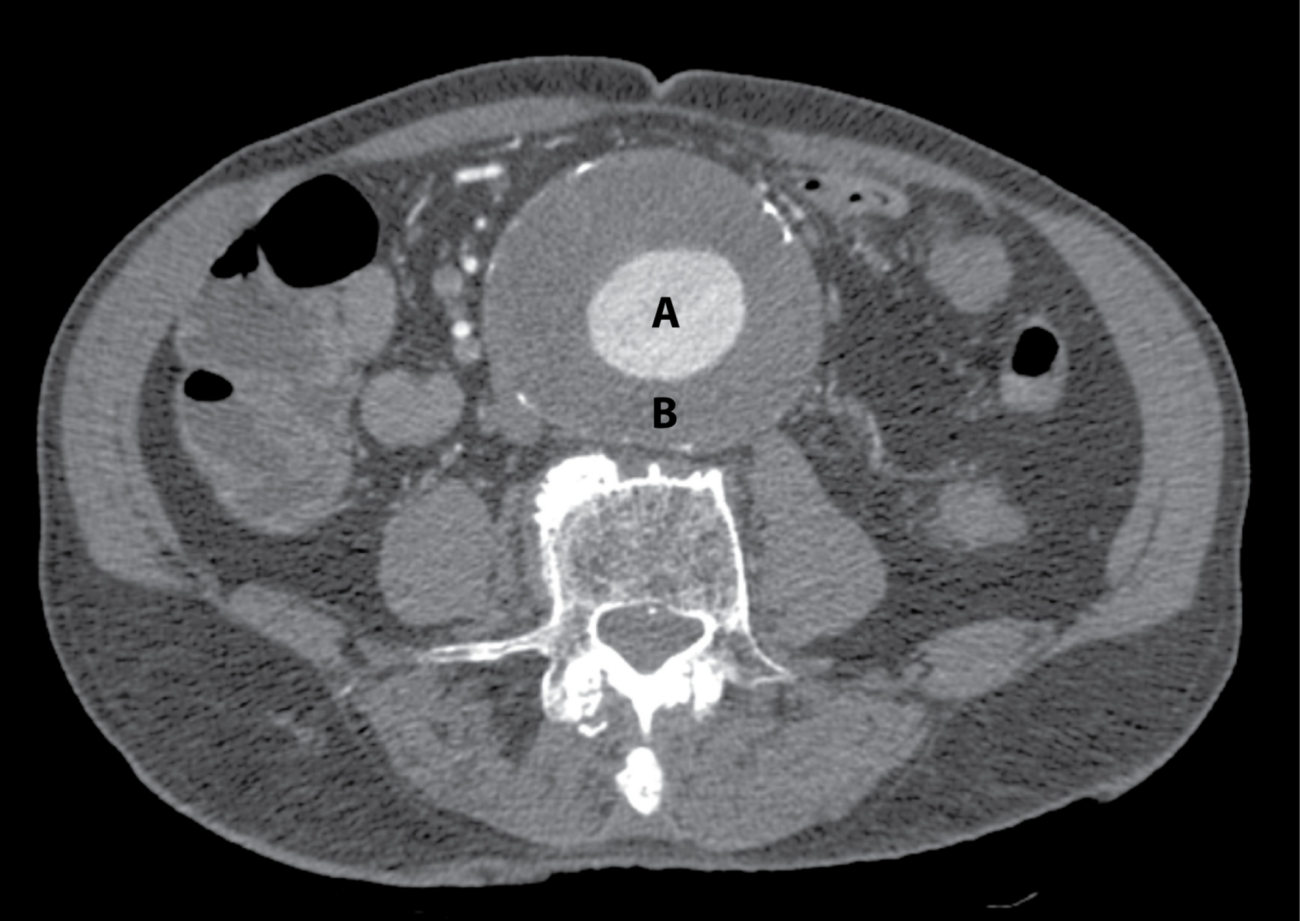
Axial computed tomography scan of the abdomen The axial computed tomography scan showed a large abdominal aortic aneurysm with a central lumen (A) and homogenous mural thrombus (B), with some calcification of the vessel wall. The contrast-enhanced images showed no extravasation or dissection and no additional useful information.

CT showed a large AAA with a homogenous thrombus surrounding a central lumen: a precarious but “stable” condition (Figure 2). The size was the same as determined with the point-of-care ultrasound.

The patient was referred to the vascular service for urgent surgical intervention.

## Discussion

As William Osler said, “There is no disease more conducive to clinical humility than aneurysm of the aorta” [4].

The prevalence of AAA is growing, especially in the elderly population, with approximately 150,000 new cases being diagnosed every year [5]. 

Most AAAs are incidentally detected and are asymptomatic [6]. The most common positive clinical finding of AAA is a pulsatile mass around the level of the umbilicus. Abdominal auscultation may reveal the presence of a bruit [7]. These findings may be incidental. A leaking or ruptured AAA is a medical emergency characterized by hypotension, shooting abdominal or back pain with or without a clinically detectable pulsatile abdominal mass. A high index of suspicion is, therefore, required for elderly patients with cardiovascular risk factors who present with hypotension and hemodynamic instability. Patients with AAA can also present with symptoms of lower limb ischemia due to embolism of thrombus from the aneurysm.

Ultrasound is the initial and preferred imaging modality used for screening and surveillance [5]. It has a sensitivity of 95% and specificity close to 100%, especially in thin patients [6]. Technical factors, such as ileus and obesity, can limit visualization, however, especially in the emergency setting. The inter-observer reproducibility of ultrasound measurements is accepted as being less than 5.0 mm. The 95% limits of agreement between ultrasound and CT measurements are usually less than the 8.0 mm difference in the anteroposterior diameter [5].

Imaging findings of aneurysm rupture include a retroperitoneal hematoma adjacent to the aneurysm as well as peri-aortic stranding. Active bleeding is indicated by the extravasation of contrast. Secondary signs of rupture include a high attenuation crescent, hematoma within either the mural thrombus or the aneurysm wall, focal discontinuity of intimal calcification, a tangential calcium sign, intimal calcification pointing away from the aneurysm, a draped aorta sign, indistinct posterior aortic wall, and the posterior aorta wall following the contour of the spine on one or both sides [6].

The presence and characteristics of the AAA intraluminal thrombus may help in identifying the risk of rupture. Ultrasound is better than CT at identifying intraluminal fissures, inhomogeneities, and dissections [3]. One of the values of ultrasound is that it provides dynamic information on organ function, in addition to information on structure. In this patient, when incorporating the ultrasound findings into the clinical decision matrix, viz. back pain, hemodynamic fragility, plus the suggestion of current deterioration within the aneurysm, urgent surgical intervention was mandated. The CT scan, however, would have been falsely reassuring if utilized in isolation.

Abdominal aortic aneurysms are one of the few surgical conditions in which size is a critical determinant of the need for intervention [5]. The risk of rupture is proportional to the size of the aneurysm and the rate of growth [6]. The rupture of AAA has a mortality rate in excess of 80% [5]. Other complications of AAA include infection, aorto-enteric and aortocaval fistulas, pseudoaneurysm, thrombotic occlusion of branch vessels, and compression of adjacent structures.

Medical treatment with statin usage as well as smoking cessation can contribute to slowing aneurysm progression [7]. Monitoring of aneurysm size and growth rate are used as indices to determine the need for elective surgical intervention [5]. The size cut-off threshold used for elective repair is an aneurysm diameter of greater than or equal to 5.5 cm. This can be via the open or endovascular approach emergency surgery. Factors that appear to impact survival include decreased time from presentation to operative intervention and the presence of a surgical team experienced in aneurysm repair [7].

Ruptured AAA can be misdiagnosed. Potential differential diagnoses include renal colic, perforated viscus, and other visceral pathologies such as diverticulitis and pancreatitis or mesenteric ischemia and aortic dissection. Patients with right-sided cardiac failure, severe tricuspid regurgitation, and hepatomegaly can also be considered [8]. Table 1 summarizes the characteristics of AAA while Table 2 highlights important differential diagnoses and their diagnostic findings.

**Table 1:** Summary table of abdominal aortic aneurysms

Etiology	Aortic dilation is a normal age-based degeneration phenomenon
Incidence	5% to 10% in all patients older than 65 years [8]
Gender Ratio	12.5% of men and 5.2% of women 74 to 84 years of age have abdominal aortic aneurysms [7]. Although a smaller percentage of women have aneurysms (approximately 20 percent of all diagnoses), women present with rupture more often than men [9]. Women rupture at a significantly smaller size than men [9].
Age predilection	Increasing incidence with increased age especially older than 65 years.
Classification	Abdominal aortic dilation of 3 cm or greater [7] Infrarenal – aneurysm originates below the renal arteries (most common) Juxtarenal – aneurysm originates at the level of the renal arteries, but the aorta at the renal arteries is normal. Pararenal – aneurysm involves the aorta at the level of the renal arteries. Suprarenal – aneurysm originates above the renal arteries.
Risk Factors	MAIN: Age (older than 65 years), male sex, smoking history. SECONDARY: family history of abdominal aortic aneurysm, coronary artery or cerebrovascular disease, hypertension, peripheral artery disease, previous myocardial infarction, hypercholesterolemia, obesity [7].
Treatment	MEDICAL: (slow aneurysm progression) smoking cessation, statin usage. SURGICAL: Elective repair – Aneurysm diameter of 5.5 cm has been used as a threshold for performing elective surgery This can be via an open or endovascular approach Emergency surgery – Factors that appear to impact survival include decreased time from presentation to operative intervention and the presence of a surgical team experienced in aneurysm repair [7].
Prognosis	Up to 50% of patients with ruptured aneurysms do not reach the hospital. Those who survive to the operating room have a mortality rate as high as 50%.
Findings on Imaging	Ultrasound: The initial and preferred imaging modality used for screening and surveillance. The presence and characteristic of an intraluminal thrombus may help in identifying the risk of rupture. Ultrasound is better than CT at identifying intraluminal fissures, inhomogeneities, and dissections [4]. CT: common imaging features of aneurysm rupture include the presence of a retroperitoneal hematoma and peri-aortic stranding. Secondary signs of rupture include high attenuation crescent, hematoma within either the mural thrombus or the aneurysmal wall, focal discontinuity of intimal calcification, tangential calcium sign, intimal calcification pointing away from the aneurysm, draped aorta sign, indistinct posterior aortic wall, posterior aorta following the contour of the spine on one or both sides. Extravasation of contrast reflects active bleeding [2,6].

**Table 2:** Differential diagnosis of abdominal aortic aneurysms

Differential diagnosis	Ultrasound evaluation	Computed tomography
Abdominal Aortic Aneurysm	Aneurysm rupture may not have associated findings besides the presence of an aneurysm larger than 5cm in diameter. Free fluid may or may not be present.	Aneurysm rupture can present with a retroperitoneal hematoma and peri-aortic stranding. Secondary signs of rupture include the crescent sign. Extravasation of contrast implies active bleeding.
Aortic dissection	Transabdominal ultrasound has a sensitivity of 70% to 80% and a specificity of 100% [8].	A dissecting membrane can be seen in the lumen of the aorta [8].
Renal calculi	Hydronephrosis may be seen. Renal calculi are not commonly visualized.	Hydronephrosis and renal calculi may be seen.
Mesenteric ischemia	Not a first-line imaging modality choice. Color Doppler and spectral waveform ultrasonography can help in evaluating the patency and adequacy of flow through the celiac and mesenteric arteries.	Focal or segmental bowel wall thickening, intestinal pneumatosis, bowel dilation, mesenteric stranding, portomesenteric thrombosis, or solid organ infarction.
Visceral pathology, e.g. diverticulitis, pancreatitis, etc.	Features specific to the affected organs.	
Enlarged liver	Diffuse liver enlargement Associated signs of heart failure, cardiomegaly, pleural effusion, pulmonary edema. Associated signs of portal hypertension, splenomegaly, portosystemic shunts [10].	

## Conclusions

While CT of AAAs is considered to be the gold standard of imaging for this pathology, ultrasound provides better information on some aspects of anatomy as well as dynamic organ function. The use of both modalities will lead to a better assessment of the acute disease process and can provide useful insight, which may ultimately affect clinical management.
